# Pre-Hospital Point-of-Care Troponin: Is It Possible to Anticipate the Diagnosis? A Preliminary Report

**DOI:** 10.3390/diagnostics15020220

**Published:** 2025-01-19

**Authors:** Cristian Lazzari, Sara Montemerani, Cosimo Fabrizi, Cecilia Sacchi, Antoine Belperio, Marilena Fantacci, Giovanni Sbrana, Agostino Ognibene, Maurizio Zanobetti, Simone Nocentini

**Affiliations:** 1 UOC Medicina d’Emergenza Urgenza e Pronto Soccorso Ospedale San Donato di Arezzo, Dipartimento Emergenza Urgenza, Azienda USL Toscana Sud-Est, 52100 Arezzo, Italymaurizio.zanobetti@uslsudest.toscana.it (M.Z.); 2 Scuola di Specializzazione in Medicina d’Emergenza Urgenza, Università degli Studi di Siena, 53100 Siena, Italy; 3 UOC Emergenza Territoriale 118 Area Provinciale Aretina, Azienda USL Toscana Sud-Est, 52100 Arezzo, Italysimone.nocentini@uslsudest.toscana.it (S.N.); 4 U.O.S.D. Analisi Chimico Cliniche, Ospedale di Nottola, Azienda USL Toscana Sud-Est, 53100 Siena, Italy; 5 UOC Elisoccorso ed Emergenza Territoriale 118 Area Provinciale Grossetana, Azienda USL Toscana Sud-Est, 58100 Grosseto, Italy; giovanni.sbrana@uslsudest.toscana.it; 6 UOC Analisi Chimico Cliniche, Ospedale San donato di Arezzo, Azienda USL Toscana Sud-Est, 52100 Arezzo, Italy; agostino.ognibene@uslsudest.toscana.it

**Keywords:** chest pain, acute myocardial infarction (AMI), acute coronary syndromes (ACSs), point-of-care testing (POCT), high-sensitivity cardiac troponin (hs-cTn), non-ST-segment elevation myocardial infarction (NSTEMI), pre-hospital

## Abstract

**Background**: Thanks to the evolution of laboratory medicine, point-of-care testing (POCT) for troponin levels in the blood (hs-cTn) has been greatly improved in order to quickly diagnose acute myocardial infarction (AMI) with an accuracy similar to standard laboratory tests. The rationale of the HEART POCT study is to propose the application of the 0/1 h European Society of Cardiology (ESC) algorithm in the pre-hospital setting using a POCT device (Atellica VTLi). **Methods**: This is a prospective study comparing patients who underwent pre-hospital point-of-care troponin testing (Atellica VTLi) with a control group that underwent standard hospital-based troponin testing (Elecsys). The primary objectives were to determine if the 0/1 h algorithm of the Atellica VTLi is non-inferior to the standard laboratory method for diagnosing AMI and to analyze rule-out/rule-in times and emergency department (ED) stay times. The secondary objective was to evaluate the feasibility of pre-hospital troponin testing. **Results**: The Atellica VTLi demonstrated reasonable sensitivity for detecting AMI, with sensitivity increasing from 60% at the first measurement (time 0) to 80% at the second measurement (time 1 h). Both the Atellica VTLi and the Elecsys method showed high negative predictive value (NPV), indicating that a negative troponin result effectively ruled out AMI in most cases. Patients in the Atellica VTLi group experienced significantly shorter times to diagnosis and discharge from the emergency department compared to the control group (Elecsys). This highlights a potential benefit of point-of-care testing: streamlining the diagnostic and treatment processes. **Conclusions**: POCT allows for rapid troponin measurement, leading to a faster diagnosis of non-ST-segment elevation myocardial infarction (NSTEMI). This enables earlier initiation of appropriate treatment, potentially improving patient outcomes and the efficiency of emergency department operations. POCT could be particularly beneficial in pre-hospital settings, enabling faster triage and transportation of patients to appropriate care centers.

## 1. Introduction

Acute coronary syndromes (ACSs) are often the first clinical manifestation of AMI with symptoms or clinical signs of myocardial ischemia, with or without modification in the 12-lead electrocardiogram (EKG) and with or without acute elevations in cardiac troponin concentrations (cTn). Patients presenting with symptoms of ACS may then be given a diagnosis of acute myocardial infarction (IMA) or unstable angina (UA) [1,2,3,4].

The symptom “chest pain” is one of the most frequent causes of visits to the emergency room (ER) (between 5 and 9%) [5], as well as one of the most common reasons for the activation of emergency medical services, and leads to high mortality (2–4%) if not diagnosed and if the patient is improperly discharged [6,7]. Patients with ACSs are difficult to diagnose due to the low specificity of the associated symptoms, which may depend on cardiac causes (with prevalence, but not exclusivity, of ACS) and non-cardiac causes (pulmonary, gastroenteric, musculoskeletal, etc.) with different incidences depending on the patient’s age group and comorbidities [8].

As reported in a meta-analysis by Fanaroff et al. [9], the features of chest pain and its associated symptoms can be used to obtain a stratification of probability but do not have significant diagnostic power. Biomarkers play a key role in the diagnosis of ACSs, stratification of risk, and management of patients with suspected ACSs. The measurement of a heart cell lesion biomarker, preferably high-sensitivity cardiac troponin (hs-cTn), is recommended in all patients with suspected ACSs. If the clinical presentation is compatible with myocardial ischemia, an increase and/or decrease in cardiac troponin above the 99th percentile of healthy individuals indicates a diagnosis of myocardial infarction according to the criteria of the fourth universal definition of myocardial infarction. In these patients, the levels of cardiac troponin increase rapidly (usually within an hour if high-sensitivity tests are used) after the onset of symptoms and remain elevated for a variable period of time (usually several days). Data from extensive multicenter studies have shown that the level of hs-cTn increases the diagnostic accuracy for myocardial infarction at the time of presentation compared to conventional testing, especially in patients presenting shortly after the onset of chest pain, allowing for a faster rule-in or rule-out of a myocardial infarction [10,11]. Overall, the tests for high-sensitivity cardiac troponin T (hs-cTnT) and high-sensitivity cardiac troponin I (hs-cTnI) appear to provide comparable diagnostic accuracy in the early detection of myocardial infarction [12]. It should always be remembered that the concentrations of hs-cTn can be affected by four variables: age, kidney dysfunction, time since the onset of chest pain, and sex [13,14]. The use of uniform cutoffs should remain the standard for the early detection of myocardial infarction. Due to their increased sensitivity and diagnostic accuracy in the detection of myocardial infarction at presentation, the time interval for the second assessment of hs-cTn can be shortened, significantly reducing the delay in diagnosis. It is recommended to use the algorithm that involves taking two samples of hs-cTn at 0/1 h or the algorithm that involves taking these samples at 0/2 h. These algorithms have been developed and validated in extensive multicenter diagnostic studies. The previously introduced ESC algorithm 0/3 h is still considered an alternative [15,16], although extensive recent trials have suggested that this protocol is less effective and less secure than the new algorithms mentioned above [17,18]. The use of POCT instrumentation, or practical devices for the bedside measurement of troponin, is a relatively recent occurrence and has undergone rapid development in recent years, even in pre-hospital settings. Currently, the use of high-sensitivity cardiac troponin POCT (hs-cTn) is increasingly widespread, as confirmed by multiple trials. For example, *Nils A Sörensen* et al. demonstrated in their study that the application of the POCT hs-cTnI method can be considered comparable to the hs-cTnI-based laboratory protocol, as recommended by international guidelines [19].

The Atellica hs-cTnI assay meets the IFCC Task Force on Clinical Applications of Cardiac Bio-Markers’ definition of a high-sensitivity troponin assay. The high-sensitivity cardiac troponin POCT has a total imprecision at the 99th percentile value of 45.20 ng/L below 10% and greater than 50% of measurements from individuals in the healthy patient population used to determine the 99th percentile value were above the Limit of Detection (LoD) of 1.60 ng/L. Its Limit of Quantitation (LoQ) is 2.50 ng/L [20,21].

## 2. Materials and Methods

The HEART POCT study was carried out in the pre-hospital setting of Arezzo (Italy) to evaluate the applicability of a 0/1 h algorithm on patients with typical chest pain with negative EKG for ST-segment elevation acute coronary syndrome (STEMI-ACS) with POCT method for troponin dosing (Atellica VTLi SIEMENS HEALTHCARE DIAGNOSTICS INC, Milano, Italy). A first hs-cTnI POCT (t_0_) was performed at the patient’s home and a second hs-cTnI POCT (t_1_) in the Emergency Department after one hour [22,23,24,25,26,27,28,29].

### Study Design

**Figure 1:**
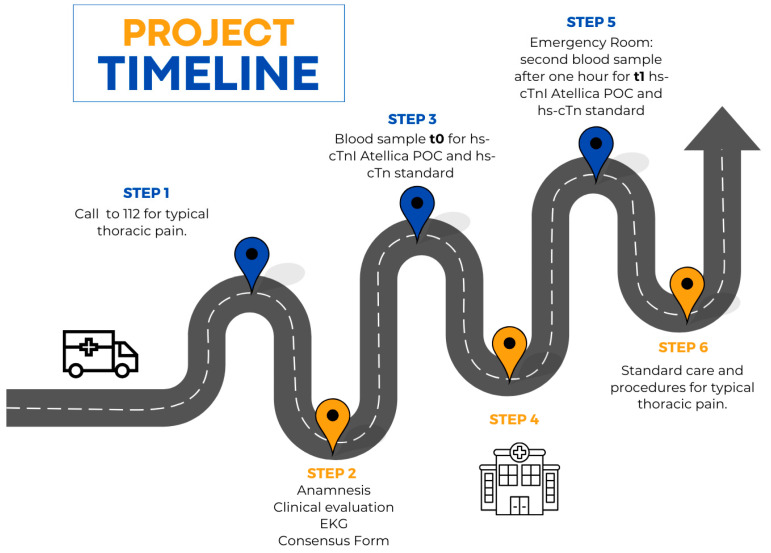
Project timeline from 112 activation to Emergency Room transport.

Patients presenting with typical chest pain and scheduled for transport by the Alfa vehicle (equipped with a medical and nursing crew) are considered eligible for study enrollment based on specific inclusion/exclusion criteria. The Alfa team conducts a medical history, performs a clinical evaluation, and acquires a 12-lead electrocardiogram (EKG). In addition to these standard procedures, a point-of-care (POC) high-sensitivity troponin I (hs-cTnI) blood sample is collected, and the patient’s risk profile is assessed using the HEART (Heart–EKG–Age–Risk Factors–Troponin) score (Figure 1). All enrolled patients are then sent to the Emergency Department, where they will perform the second dosage of hs-cTn POCT at a distance of 1 h from the troponin performed in pre-hospital and will be managed according to the 0/1 h protocol. Finally, the times of the patients in ED will be recorded.

Heart POCT is a preliminary, single-center clinical trial currently limited by a small sample size (enrollment began in June 2023) and the lack of established cut-off values in the literature for the 0/1-h protocol using the Atellica^®^ (SIEMENS HEALTHCARE DIAGNOSTICS INC, Milano, Italy) VTLi troponin assay.

***The primary aims of this study are:***

- To evaluate the non-inferiority of the 0/1-h ESC algorithm using Atellica hs-cTnI point-of-care (POC) testing compared to the conventional laboratory technique (hs-cTnT Elecsys®, Roche, Basel, Switzerland) for the early diagnosis of acute myocardial infarction (AMI). A non-inferiority margin of 5% difference in negative predictive value is defined. This aims to determine if the new POC test can effectively rule out AMI with a probability at least equivalent to the standard laboratory test, considering the initial sample collection at the patient’s location followed by a second sample in the Emergency Department (ED).
- To analyze rule-out/rule-in times for non-ST-elevation acute coronary syndromes (NSTE-ACS), as well as ED stay times, and correlate these with corresponding times obtained using the standard 0/3-h intra-hospital protocol (involving laboratory-based hs-cTnT testing).

***The secondary aims of the study are:***

- Evaluate the feasibility of the first hs-cTn POCT sampling performed in the pre-hospital environment, with the instrumentation provided.

***Study population and recruitment criteria:***

***Inclusion criteria:***

- over 18 years of age;
- acute non-traumatic chest pain;
- informed consent obtained from the patient.

***Criteria for exclusion:***

- less than 18 years of age;
- pregnant women;
- SARS-CoV-2-positive patients;
- patients with ST-elevation myocardial infarction EKG;
- hemodynamically unstable patients.

To evaluate non-inferiority, 36 patients were enrolled and tested with both Atellica hs-cTnI POC and hs-cTnT Elecsys® Roche. The Elecsys^®^ Roche assay has a measuring range of 3–10,000 ng/L, a limit of quantification (LoQ) of 13 ng/L, a limit of detection (LoD) of 3 ng/L, and an upper reference limit (URL) of 14 ng/L (95% confidence interval: 12.7–24.9 ng/L) [30]. All enrolled patients had activated the Emergency Health Service (by calling 112) for acute chest pain of non-traumatic origin from June 2023. 

The study group consists of 36 patients: 20 males (average age 62 years, range 37–90 years) and 16 females (average age 72 years, range 51–94 years). Table 1 summarizes the characteristics of the study population.

To analyze the rule-out/rule-in times for NSTEMI, as well as the ED stay times, and correlate them with those obtained using the standard intra-hospital protocol (which involves laboratory dosing of hs-cTnT Elecsys^®^ with a 0/3-h algorithm), we compared the first group of 36 patients with a control group of 61 patients (41 males and 20 females). The control group had similar selection criteria to the Atellica group:

- Same activation code: (patients were transported to the ER of Arezzo without Atellica VTLi) during the same reference period (June–September 2023).
- Similar inclusion/exclusion criteria.
- Stratification based on cardiovascular risk factors and HEART score.

The key difference between the groups was the application of a protocol entirely within the hospital, with hs-cTnT Elecsys^®^ detection performed in the laboratory using the 0/3 h algorithm. The characteristics of the Elecsys control group are listed in Table 2.

The observed difference in sample size between the two groups was primarily due to limited patient availability at the recruitment center, as only one Atellica VTLi device was available. Despite this, a thorough analysis of baseline characteristics between the groups revealed no significant differences that could potentially impact the study outcomes. 

## 3. Results

First of all, the absence of studies that currently validate the 0/1 ESC algorithm with Atellica VTLi has required us to process simultaneously the same blood samples in the laboratory, looking for a correlation between the values of hs-cTnI and hs-cTnT derived from the two methods (Table 3).

Despite the limitation of the small sample size in the current study, a substantial overlap was observed between the values obtained for the first sample (time 0) and the second sample (time 1 h). No statistically significant difference between the two techniques was identified, according to Student’s *t*-tests for paired data: *p*-value = 0.1940 for hs-cTn comparisons between Atellica and Roche at time 0, and *p*-value = 0.1765 for the respective values at time 1 h. Of the total population (36 patients), 26 were discharged home based on two consecutive negative Atellica values (consistent with rule-out according to both the 0/1-h laboratory protocol and the POC protocol). Two of these patients, with symptom onset exceeding 3 h, exhibited initial hs-cTnI values of 4 ng/L. According to current best practices for Atellica VTLi (Table 1), these patients could have been eligible for early rule-out based on a single measurement. 

In 100% of the discharged subjects, the 6-week follow-up was negative for major adverse cardiac events (MACEs), supporting the safety of early rule-out in these patients. However, in the remaining 10 patients, troponin elevation compatible with myocardial damage was observed by at least one method (POC or traditional laboratory). Of these ten patients, five received a definitive diagnosis of non-ST-elevation myocardial infarction (NSTEMI) (confirmed by coronary angiography and treated accordingly). The remaining five patients were subsequentlhy diagnosed with myocarditis (*n* = 1), pulmonary embolism (*n* = 1), heart failure (*n* = 2), and pericardial metastases in a patient with lymphoma (*n* = 1). Using the Upper Reference Limit (URL) of the 99th percentile as a cut-off for NSTEMI diagnosis, Atellica VTLi demonstrated a sensitivity of 60% (95% CI: 17.1–100%) and a Negative Predictive Value (NPV) of 92.9% (95% CI: 83.3–100%) at time 0. At time 1 h, sensitivity increased to 80% (95% CI: 44.9–100%) and NPV to 93.3% (95% CI: 89.2–100%). In contrast, analysis of the same samples in the laboratory (interpreted according to the 0/1-h ESC protocol) yielded both sensitivity and NPV of 100%, albeit with a small sample size. The difference between Roche and Atellica was observed in a single patient. This patient, with hs-cTnI values below the 23 ng/L cut-off at both time 0 (15 ng/L) and time 1 h (20.3 ng/L), was not identified by Atellica VTLi using the 99th percentile URL. However, laboratory measurements for this patient were 21 ng/L at time 0 and 32 ng/L at time 1 h, leading to a rule-in decision according to the 0/1-h ESC protocol with its specific cut-offs for hs-cTnT Elecsys Roche.

This discrepancy highlights the previously discussed limitation: the lack of studies currently validating the 0/1-h ESC algorithm with Atellica VTLi. 

To compare rule-in/rule-out times for access to the hemodynamics room and hospital discharge, a control group (Elecsys group) was included. This group, consisting of 61 patients, underwent hs-cTnT testing according to the standard 0/3-h hospital protocol. Of the Elecsys group, 11 patients were ruled-in for NSTEMI and underwent hemodynamic evaluation (all with positive coronary angiography). Among the remaining fifty patients, forty-four were discharged home, while six were admitted for alternative diagnoses such as pneumonia (*n* = 1), unstable angina (*n* = 3), and heart failure (*n* = 2). 

Comparing rule-in times for access to the hemodynamics room between the Atellica and Elecsys groups revealed a statistically significant difference (paired *t*-test, t-value 4.863, *p*-value = 0.0083). The mean Emergency Department (ED) residence time for rule-in patients was significantly shorter in the Atellica group (60.8 min, SD 9.58 min) compared to the Elecsys group (281.4 min, SD 93.25 min), as shown in Table 4. 

Similarly, a statistically significant difference was observed between the two groups regarding rule-out times for NSTEMI in discharged patients (paired *t*-test, t-value 6.409, *p*-value < 0.0001). The mean Emergency Department (ED) stay time for discharged patients was significantly shorter in the Atellica group (174.8 min, SD 126.7 min) compared to the Elecsys group (322.5 min, SD 73.94 min), as shown in Table 5.

## 4. Discussion

Based on preliminary data and our experience, the use of Atellica VTLi in the pre-hospital setting for the early diagnosis of NSTEMI appears feasible and compatible with existing pre-hospital management protocols. The comparison of hs-cTnI values obtained from Atellica VTLi with those from the Elecsys-Roche laboratory method demonstrated a substantial correlation, suggesting that the point-of-care technique is comparable in accuracy to the conventional laboratory method.

However, while a single cut-off of hs-cTnI equal to the 99th percentile of the healthy population URL [31] has been previously considered for Atellica VTLi, utilizing this technique within a 0/1 h rapid algorithm requires further investigation. Studies with larger populations are necessary to establish optimal cut-off values for the Atellica VTLi hs-cTnI assay and enhance its diagnostic accuracy, similar to recent studies (e.g., Cullen L. et al., 2024 [26]) that evaluated 0/2 h algorithms. These future studies should aim to establish troponin values (including spot values and variations) that achieve an NPV of 99% and a positive predictive value (PPV) of at least 70% for acute myocardial infarction, aligning with the accuracy and safety criteria outlined in ESC guidelines. The observed reduction in time to diagnosis and emergency department stay for NSTEMI can be attributed to several factors:

- Adoption of a faster algorithm: The 0/1 h algorithm used with Atellica VTLi is significantly faster than the traditional 0/3 h algorithm.
- Pre-hospital blood sampling: Obtaining the first blood sample before hospital arrival accelerates the diagnostic process.
- Rapid processing time: Atellica VTLi provides results much faster than traditional laboratory analysis (7.5 min compared to 45–60 min).

These improvements in efficiency translate into numerous benefits for both patients and the healthcare system, including:

- Early identification and treatment of NSTEMI.
- Reduced time spent in the emergency department.
- Potential for improved patient outcomes.
- Reduced healthcare resource utilization and associated costs.

As regards the evaluation of the feasibility of this POCT device in the pre-hospital setting, we can record the same problem already highlighted in other cases: the susceptibility to vibrations. We tried to overcome the problem by taking some precautions:

- Stable surface: The Atellica VTLi was always placed on a stable, level surface during operation to minimize the effects of external vibrations. We did not analyze the sample in the ambulance.
- Operator training: Operators were thoroughly trained on proper handling and placement of the device to ensure minimal errors during sample processing.
- Monitoring of device stability: The stability of the device was monitored throughout the study by laboratory experts.

## 5. Conclusions

The widespread diffusion of the POCT technique in Emergency Rooms could allow a faster diagnosis of NSTEMI and an earlier initiation of appropriate treatment, potentially improving patient outcomes and patient care. Point-of-care testing could streamline the diagnostic process, reducing the time required for laboratory testing and result reporting. The potential reduction in hospital stays and the potential improvement of resource utilization could translate into cost savings for the healthcare system and reduce hospital congestion. From the previous observations, an area of future discussion could be that of cost-effectiveness. An economic analysis comparing the costs associated with each testing strategy would be valuable. This should consider the cost of the point-of-care device, consumables, labor, and the impact on hospital resource utilization.

The widespread diffusion of the POCT technique on vehicles equipped with healthcare personnel and operating in isolated areas with hospitals without hemodynamic units would ensure a more suitable primary centralization of patients at risk to a hospital with hemodynamic units. This strategy could reduce the time to treatment, allowing the patient to reach the appropriate setting for the right therapy in the shortest possible time.

It could also be interesting to investigate in future studies how the immediate result of hs-cTn POCT influences clinical decision making among emergency physicians and how it could impact on the patient’s level of anxiety.

The findings of this study may not be generalizable to all patient populations due to the relatively small sample size and the specific characteristics of the study population. Further studies in larger and more diverse populations are needed.

## Figures and Tables

**Table 1:** Characteristics of the population enrolled with Atellica VTLi.

	Total Patients (*n* = 36)	Males (*n* = 20)	Females (*n* = 16)
Age [age: average ± Standard deviation (DS)]	66.78 ± 14.29	62.05 ± 14.03	72.69 ± 13.17
Hypercholesterolemia	13 (36%)	8 (40%)	5 (38%)
Arterial hypertension	25 (69%)	15 (75%)	10 (63%)
Diabetes mellitus	8 (22%)	4 (20%)	4 (25%)
Obesity	6 (17%)	3 (15%)	3 (19%)
Active smoking	6 (17%)	2 (10%)	4 (25%)
Familiarity for Coronary Artery Dissease (CAD)	5 (14%)	3 (15%)	2 (13%)
Atherosclerotic pathology *	11 (31%)	6 (30%)	5 (38%)
HEART score			
High risk (score 7–10)	7 (19%)	2 (10%)	5 (31%)
Medium risk (score 4–6)	18 (50%)	12 (60%)	6 (38%)
Low risk (score 0–3)	11 (31%)	6 (30%)	5 (31%)

* Atherosclerotic pathology includes previous AMI/Percutaneous Coronary Intervention (PCI)/Coronary Artery Bypass Graft (CABG), Transient Ischemic Attack (TIA)/stroke or peripheral arterial disease.

**Table 2:** Characteristics of the control population sampled with Elecsys, Roche.

	Total Patients (*n* = 61)	Males (*n* = 41)	Females (*n* = 20)
Age (ages: averages ± DS)	67.56 ± 12.92	65.46 ± 12.29	71.85 ± 13.74
Comorbidity			
Hypercholesterolemia	26 (43%)	21 (51%)	5 (25%)
Arterial hypertension	43 (70%)	30 (73%)	13 (65%)
Diabetes mellitus	13 (21%)	7 (17%)	6 (30%)
Obesity	9 (15%)	5 (12%)	4 (20%)
Active smoking	15 (25%)	13 (32%)	2 (10%)
Familiarity for CAD	9 (15%)	7 (17%)	2 (10%)
Atherosclerotic pathology *	25 (41%)	21 (51%)	4 (20%)
HEART score			
High risk (score 7–10)	7 (19%)	2 (10%)	5 (31%)
Medium risk (score 4–6)	18 (50%)	12 (60%)	6 (38%)
Low risk (score 0–3)	11 (31%)	6 (30%)	5 (31%)

* Atherosclerotic pathology includes previous IMA/PCI/CABG, TIA/stroke or peripheral arterial disease.

**Table 3:** hs-cTn values (expressed in ng/L) obtained from analysis of the same blood samples taken at 0/1 h times by POCT (hs-cTnI Atellica) and laboratory (hs-cTnT Roche).

hs-cTnI Atellica t 0 h	hs-cTnT Roche t 0 h	hs-cTnI Atellica t 1 h	hs-cTnT Roche t 1 h
8.1	9	7.0	6
11.9	13	11.2	16
5.5	7	7.3	14
6.4	7	9.6	16
3.0	5	3.5	4
3.9	4	4.2	6
9.1	10	7.0	8
47.8	80	44.6	74
8.7	11	8.2	8
14.3	27	15.0	27
5.0	4	5.0	5
15.2	21	16.4	22
19.0	17	18.2	17
11.2	12	12.0	14
1250.0	505	1125.0	480
6.0	7	5.7	8
9.0	4	8.1	5
9.7	9	10.3	8
24.0	18	26.0	19
22.0	16	20.0	15
21.0	17	21.8	15
661.0	190	600.0	210
8.1	9	4.0	10
8.1	8	6.5	9
15.9	7	14.5	9
36.1	19	65.3	53
15.0	21	20.3	32
12.9	19	15.0	18
11.0	14	18.0	16
15.7	25	40.0	85
153.0	101	498.0	213
4.6	11	6.4	12
9.1	10	5.7	9
9.2	15	8.2	14
31.6	80	31.6	81
32.7	47	81.0	140

**Table 4:** *t*-tests with paired samples on hemodynamics admission times in patients with rule-in in the two groups (Atellica and Elechsys).

Group	Atellica Acc-Ric	Elecsys Acc-Ric
Mean	60.8	281.4
SD ^a^	9.58	93.25
SEM ^b^	4.28	41.7
Mean.Diff ^c^	220.6	
DF ^d^	4	
t-value	4.863	
*p*-value	0.0083	

SD ^a^: standard deviation, SEM ^b^: standard error of the mean, Mean.Diff ^c^: mean difference, DF ^d^: degree of freedom.

**Table 5:** *t*-tests with paired samples on discharge times in patients who have gone through rule-outs in the two groups (Atellica and Elecsys).

Group	Atellica Acc-Dim	Roche Acc-Dim
Mean	174.81	322.52
SD ^a^	126.7	73.94
SEM ^b^	22.76	13.28
Mean.Diff ^c^	147.71	
DF ^d^	30	
t-value	6.409	
*p*-value	<0.0001	

SD ^a^: standard deviation, SEM ^b^: standard error of the mean, Mean.Diff ^c^: mean difference, DF ^d^: degree of freedom.

## Data Availability

The data presented in this study are available on request from the corresponding author due to privacy.
